# THE ESSENTIAL ROLE OF NURSES IN INFLAMMATORY BOWEL DISEASE MANAGEMENT: A MULTIDISCIPLINARY APPROACH IN A COLOMBIAN IBD CENTER

**DOI:** 10.1590/S0004-2803.24612025-044

**Published:** 2026-03-02

**Authors:** Ginary ORDUZ-DÍAZ, Viviana PARRA-IZQUIERDO, Andrea REATIGA, Oscar Mariano PINTO, Juliep SARMIENTO, Carlos CUADROS-MENDOZA, Johon GARCES-CAMACHO, Samuel CUBILLOS-RODRIGUEZ, Cristian FLÓREZ-SARMIENTO, Juan Sebastián FRÍAS-ORDOÑEZ

**Affiliations:** 1 Hospital Internacional de Colombia, Nurse of the Centre of excellence in inflammatory bowel disease, Bucaramanga, Colombia.; 2 Hospital Internacional de Colombia, Centre of excellence in inflammatory bowel disease, Bucaramanga, Colombia.; 3 El Bosque University, Cellular and molecular immunology group, Bogota, Colombia.

**Keywords:** Inflammatory bowel diseases, nurses, patient education as topic, fatigue, multidisciplinary care team, Doenças inflamatórias intestinais, enfermeiros, educação do paciente como assunto, fadiga, equipe de assistência ao paciente

## Abstract

**Background:**

Inflammatory bowel disease (IBD) requires a multidisciplinary approach due to its complexity. Nurses play a key role in disease management, patient education, and care coordination. This study describes the role of nurses in an IBD Center of Excellence in Colombia, focusing on five pillars: clinical care, research, quality of life (including fatigue and mental health assessment), empowerment, and multidisciplinary support.

**Methods:**

Descriptive observational study conducted between 2023 and 2024, using semi-structured interviews, direct observations, and surveys with the nursing team. Data were analyzed using descriptive statistics and thematic analysis. Measures were taken to minimize observer and interviewer bias.

**Results:**

A total of 90 IBD patients were evaluated (56.6% female; mean age 40.8 years). Nurses played a central role in patient education, phenotyping, follow-up, and performing intestinal ultrasound. The IBDQ-32 questionnaire revealed moderate impact on quality of life, and the IBD-F scale identified fatigue in a subset of patients despite clinical remission. Patient satisfaction remained above 98% in both years. Nurses also coordinated pediatric-to-adult transitions, organized multidisciplinary meetings, and contributed to 41 research posters, some of which received international recognition.

**Conclusion:**

Nurses are essential in IBD management, contributing to clinical care, education, quality of life assessment, research, and patient empowerment. Their involvement enhances outcomes, satisfaction, and the efficiency of multidisciplinary teams. Expanding standardized nurse training in Latin America is vital to strengthen IBD care.

## HIGHLIGHTS

• Nurses are pivotal in multidisciplinary IBD management.

• They lead patient education, follow-up, and intestinal ultrasound.

• Quality of life and fatigue were systematically assessed.

• Patient satisfaction consistently exceeded 98%.

• Expanding nurse training enhances IBD care in Latin America.

## RESUMO

**Contexto:**

A Doença Inflamatória Intestinal (DII) exige uma abordagem multidisciplinar devido à sua complexidade. Os enfermeiros desempenham um papel fundamental no manejo da doença, na educação dos pacientes e na coordenação do cuidado. Este estudo descreve o papel dos enfermeiros em um Centro de Excelência em DII na Colômbia, com foco em cinco pilares: atendimento clínico, pesquisa, qualidade de vida (incluindo avaliação da fadiga e da saúde mental), empoderamento e apoio multidisciplinar.

**Métodos:**

Estudo observacional descritivo realizado entre 2023 e 2024, utilizando entrevistas semiestruturadas, observações diretas e questionários com a equipe de enfermagem. Os dados foram analisados por meio de estatísticas descritivas e análise temática. Medidas foram adotadas para minimizar vieses do observador e do entrevistador.

**Resultados:**

Um total de 90 pacientes com DII foi avaliado (56,6% mulheres; média de idade de 40,8 anos). Os enfermeiros desempenharam um papel central na educação, fenotipagem, seguimento clínico e realização de ultrassonografia intestinal. O questionário IBDQ-32 revelou impacto moderado na qualidade de vida, e a escala IBD-F identificou fadiga em uma parcela dos pacientes, mesmo em remissão clínica. A satisfação dos pacientes permaneceu acima de 98% em ambos os anos. Os enfermeiros também coordenaram a transição de pacientes pediátricos para o atendimento adulto, organizaram reuniões multidisciplinares e contribuíram com 41 pôsteres de pesquisa, alguns dos quais receberam reconhecimento internacional.

**Conclusão:**

Os enfermeiros são essenciais no tratamento da DII, contribuindo para os cuidados clínicos, a educação, a avaliação da qualidade de vida, a pesquisa e o empoderamento dos pacientes. Sua atuação melhora os desfechos, a satisfação e a eficiência das equipes multidisciplinares. A ampliação da formação padronizada em enfermagem na América Latina é fundamental para fortalecer o cuidado em DII.

## INTRODUCTION

The management of inflammatory bowel disease (IBD) presents several challenges due to its chronic nature, variable presentation, and the need for a comprehensive, multidisciplinary approach^1^ ^-^ ^3^. One of the primary difficulties lies in the heterogeneity of disease manifestations, which may include complications such as strictures, fistulas, or colorectal cancer. These clinical variations demand early diagnosis and personalized treatment strategies tailored to each patient’s disease phenotype and severity^1^. As in other immune-mediated inflammatory conditions, optimal care extends beyond pharmacological interventions to include lifestyle modifications, psychological support, and coordinated multidisciplinary input^2^ ^-^ ^4^.

**FIGURE 1:**
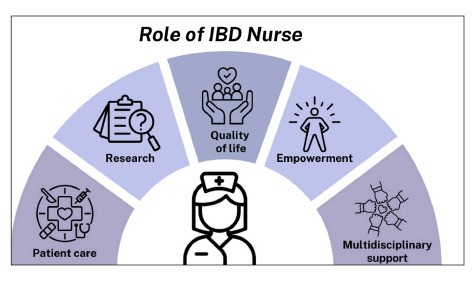
Core domains of nursing practice in inflammatory bowel disease management. Source: authors.

The active participation of nursing professionals in the management of patients with IBD is driven by the increasing recognition of the disease’s complexity and the limitations of physician-centered care models. Nurses are key contributors to high-quality care, acting as coordinators, educators, and advocates for patients (Figure 1). Evidence has demonstrated that the inclusion of dedicated IBD nurse specialists improves outcomes, reduces emergency department visits, and facilitates faster access to care^5^ ^-^ ^8^. Their presence also contributes to cost-effective management by decreasing hospitalizations and enhancing outpatient care delivery.

Furthermore, IBD-specialized nurses play a critical role in addressing issues often underrecognized in routine clinical assessments, such as fatigue, anxiety, and depression. They support disease monitoring, educate patients on therapeutic regimens, and coordinate transitions from pediatric to adult care, thereby improving continuity and adherence^9^ ^-^ ^13^. Their role is particularly valuable in Latin America, where few centers have formal IBD nurse programs and standardized training remains scarce^11^ ^,^ ^14^. In these contexts, exploring the perceptions, functions, and contributions of nursing professionals is essential for optimizing care models.

This study aims to describe the role of the nurse in IBD management within a Colombian referral center, focusing on key domains such as clinical care, research, fatigue and mental health assessment, patient empowerment, and multidisciplinary collaboration. In view of the qualitative components of this research, including direct observation and semi-structured interviews, efforts were made to minimize interviewer or observer influence. These included using standardized interview guides, ensuring data triangulation, and maintaining analytic neutrality during the thematic analysis process.

## METHODS

### Study design

This was a prospective, descriptive observational study with mixed-methods components, conducted at the IBD Center of Excellence of the Hospital Internacional de Colombia (HIC) between January 2023 and March 2024. The aim was to characterize and analyze the role of specialized nurses in the comprehensive management of IBD patients. The study was longitudinal, following patients and nursing practices over a 12-month period. Although educational interventions were included (e.g., care modules and symptom monitoring materials), these were already part of standard institutional practice and were not modified or introduced for research purposes; thus, the study design remained observational.

### Extended description of study subjects

In addition to the IBD patients, this study included the IBD nurse as a subject of observation and interview. The nurse’s perspectives, practices, and experiences were evaluated as part of the qualitative component of the research. Her inclusion aimed to capture in-depth insights into nursing contributions and workflows, using structured observation and semi-structured interviews.

### Inclusion and exclusion criteria

We included:

- Patients diagnosed with ulcerative colitis or Crohn’s disease.
- Adults and pediatric patients aged ≥7 years.
- Patients receiving care from the IBD nurse within the study period.

We included:

- Patients who did not complete at least two follow-up interactions with the IBD nurse.
- Patients with incomplete clinical records.
- Patients with cognitive impairments limiting questionnaire participation.

### Nurse participant inclusion and exclusion criteria

We included:

- The certified IBD nurse working at the center during the study period.
- Actively engaged in patient care, education, and coordination for ≥12 months.
- Willing to participate in structured interviews and observations.

We excluded:

- Nursing staff not specialized in IBD care.
- Temporary staff or those with less than six months of clinical interaction with IBD patients.

The nurse provided written informed consent for participation in the research as a study subject, distinct from her clinical duties. 

### Sample size and setting

A total of 90 patients were included. The sample was not calculated statistically as this was an exploratory, descriptive study based on the total number of patients actively followed by the IBD nurse during the 12-month period. Data were collected through structured observation and interviews conducted in both the outpatient IBD clinic and inpatient hospitalization units, ensuring a comprehensive view of nursing practice across care settings.

### Data collection procedures

Data collection involved:

- Direct observation of nurse-patient interactions, care activities, and interprofessionalmeetings.
- Semi-structured interviews with the IBD nurse to explore perceptions, processes, and interventions.
- Document review (activity logs, educational materials, and institutional protocols).
- Patient-reported outcomes using validated instruments.

To minimize observer and interviewer bias, all interviews followed a standardized guide developed by the research team. Observers were trained to record behaviors objectively. Data triangulation was used, comparing nurse responses, observations, and patient records. Interviews were conducted in private settings to avoid social desirability bias.

A semi-structured interview guide was specifically developed for this study by the research team, based on the key domains of nursing practice in IBD described in international literature. The instrument was designed to explore the nurse’s roles, challenges, perceptions, and contributions to patient care, education, research, and multidisciplinary coordination. The guide consisted of 12 open-ended questions organized across five thematic categories: clinical care, patient education, psychosocial support, quality indicators, and institutional processes. This guide is provided as SUPPLEMENTARY MATERIAL S1.

**Supplementary Material S1**

**Semi-Structured Interview Guide for the IBD Nurse**

This interview guide was developed by the research team to explore the nurse’s experience and role in IBD care. It includes open-ended questions covering clinical responsibilities, patient education, care coordination, challenges, empowerment, and research contributions. The guide was validated by two senior IBD specialists and piloted before use.

**Domain 1 - Clinical Care**

1. Can you describe your main clinical responsibilities in managing IBD patients?

2. How do you participate in disease activity monitoring (e.g., symptoms, flares)?

3. What is your role in coordinating care with physicians and other specialists?

**Domain 2 - Patient Education and Empowerment**

4. What educational interventions do you provide to newly diagnosed patients?

5. How do you promote patient self-management and adherence?

**Domain 3 - Psychosocial and Emotional Support**

6. What challenges do you observe regarding patients’ emotional well-being?

7. How do you support patients facing anxiety, depression, or fatigue?

**Domain 4 - Quality of Care and Institutional Involvement**

8. How do you contribute to institutional indicators and quality monitoring?

9. Have you participated in initiatives to improve clinical protocols or patient flow?

**Domain 5 - Research and Professional Development**

10. Have you been involved in research or academic activities related to IBD?

11. How has your role evolved through training and continuing education?

12. What challenges or opportunities do you see for nursing in IBD care in Latin America?

### Instruments and theoretical framework

Three validated self-report tools were used in this study: the Inflammatory Bowel Disease Questionnaire (IBDQ-32) the Inflammatory Bowel Disease Fatigue Scale (IBD-F)^9^ ^,^ ^15^ ^-^ ^17^, and the Hospital An­xiety and Depression Scale (HADS)^14^ ^,^ ^18^. These instruments were selected based on their psychometric validity, wide use in IBD populations, and relevance to nur­sing care.

### Nurse training

The IBD nurse had formal certification in IBD management, obtained through structured programs by the Colombian Association of Gastroenterology and the Pan American Crohn’s and Colitis Organization (PANCCO). Training included:

- Duration: 6 months
- Content: Pathophysiology, diagnostic criteria, pharmacological and non-pharmacological treatment, psychosocial aspects, nutritional management, care of pregnant patients with IBD, and transitional care.
- Schedule: Weekly 2-hour sessions, supervised by IBD specialists.
- Methodology: Lectures, case-based discussions.
- Evaluation: Discussions, write and publish an article.

Practical training for 2 weeks in Hospital La Fe of Valencia, Spain, and UZ Leuven in Belgium.

Practical intensive training for IBD specialists at the International Hospital of Colombia, for 3 months.

### Ethical considerations

The study was approved by the Institutional Ethics Committee of HIC (approval code: CEI-2024-08988) and conducted in accordance with the principles of the Declaration of Helsinki. All participants gave informed consent, and confidentiality was strictly maintained. The IBD nurse involved in the study also participated as a research subject in the qualitative component. Her consent was obtained separately, and measures were taken to ensure confidentiality and prevent conflicts of interest between her dual roles as participant and provider.

### Ethical Statement

This research was reviewed and approved by the institutional review board and ethics committee of each institution. Its design took into account the requirements established in the Declaration of Helsinki, 2013 version, Fortaleza, Brazil, and was considered a low-risk research. Confidentiality of the information collected from patients was guaranteed.

## RESULTS

### Sociodemographic and professional profile of the nurse

A single certified IBD nurse participated in this study. She was a female professional with over seven years of experience in internal medicine and chronic disease care, who had completed a six-month structured training program with the Colombian Association of Gastroenterology and PANCCO, complemented by international rotations with 3 months and half practical training at Hospital La Fe of Valencia, UZ Leuven and International Colombian Hospital. Quarterly continuing education sessions were provided by physicians, and peer-to-peer case discussions allowed for nurse-led training. Participation in national and international congresses enhanced the nurse’s knowledge and leadership role.

### Patient characteristics

Ninety patients with IBD were included (56.6% female; mean age: 40.8 years; range: 7-80), including ten pediatric patients. Ulcerative colitis (UC) accounted for 68.8% of cases, while Crohn’s disease (CD) represented 31.1%. Phenotypic classifications and treatment regimens are summarized in Table 1. Biologic therapy was used by 27.4% of UC and 60.7% of CD patients (Table 2).

**TABLE 1:** Characteristics of patients included in follow-up by nurses at the IBD Centre of Excellence (n=90).

Number of patients (n=90)	Ulcerative colitis (n=62)	Crohn’s Disease (n=28)
Age (promedium), years	40.8	
Sex		
Female (%)	56.6%	
Male (%)	43.3%	
Localization		
Pancolitis (%)	37%	
Left colitis (%)	54.8%	
Proctitis (%)	8%	
Ileal (%)		64.2%
Ileo-colonic (%)		28.5%
Colonic (%)		7.14%
Flare hospitalization (%)	27.7%	28.5%
Biologic therapy (%)	27.4%	60.7%

Source: Authors

**TABLE 2:** Biologic therapy in IBD patients at the IBD centre of excellence in 2023 and 2024.

Biological drug	Ulcerative Colitis (n=62)	Crohn´s Disease (n=28)
Infliximab (%)	47%	45%
Adalimumab (%)	25.5%	32.4%
Vedolizumab (%)	23.5%	19.2%
Golimumab (%)	5.8%	2.7%

Source: Authors

### Educational interventions

A modular educational program was delivered to each newly diagnosed patient, covering IBD generalities, symptom monitoring, nutrition, lifestyle, and pregnancy. The program included five audiovisual modules, totaling approximately five hours per patient. Evaluations were conducted post-module with patients and caregivers. Additionally, bimonthly ludo-educational hospital rounds attracted over 350 participants, enhancing awareness and community engagement.

### Telecommunication and follow-up

**FIGURE 2:**
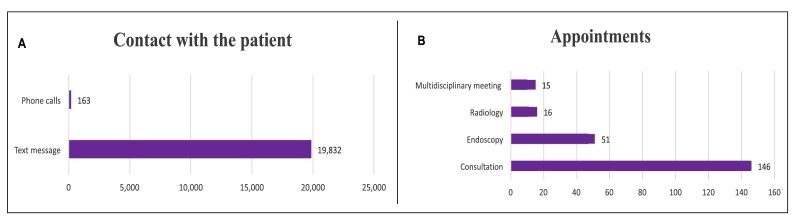
**A)** contact with the patients. **B)** appointments. Source: authors

A 24/7 phone line managed by the nurse provided access to both adult and pediatric patients. Between January 2023 and September 2024, the center received 19,832 instant messages and 163 phone calls (Figure 2A). Scheduling via email included 146 consultations, 51 endoscopic exams, 16 imaging studies, and 15 multidisciplinary meetings (Figure 2B).

### Intestinal ultrasound

Intestinal ultrasound is routinely performed at the institution as part of the standard care pathway. During scheduled IBD ultrasound events, the nurse coordinated patient triage based on clinical urgency and disease activity, ensuring optimal workflow and collaboration with gastroenterologists.

### Quality of life assessment (including fatigue and mental health): IBDQ-32

**FIGURE 3:**
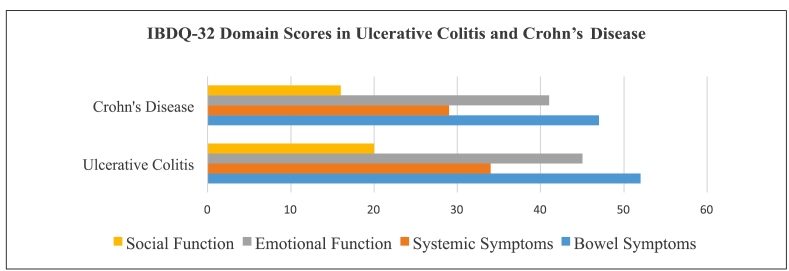
Comparison of IBDQ-32 domain scores between patients with ulcerative colitis and crohn’s disease. Source: authors.

The IBDQ-32 was self-administered by 56 adult patients after clinical visits, while pediatric patients were excluded due to lack of validation. The median score was 150 (IQR: 118.3-181.5), with lower scores in CD (median: 133) than UC (median: 151), though differences were not statistically significant. The most impaired domain was systemic symptoms, reflecting a significant impact on daily functioning and emotional well-being. Results are presented in Figure 3 **,** statistically significant differences were observed in bowel symptoms (*P*=0.04), systemic symptoms (*P*=0.03), and social function (*P*=0.05), with Crohn’s Disease patients reporting lower scores in all domains.

### Quality indicators and performance metrics

**FIGURE 4:**
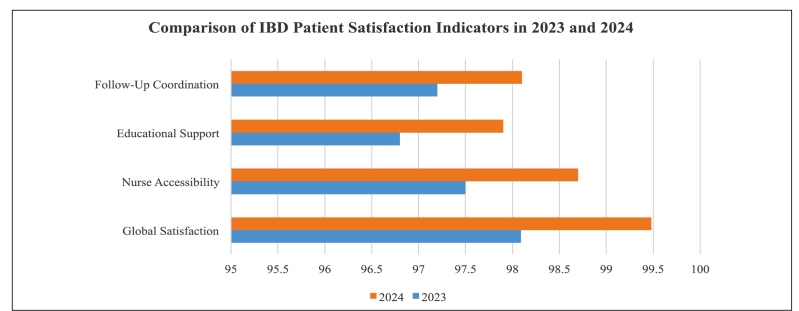
Year-to-Year Improvement in IBD Patient Satisfaction (2023-2024). Source: authors.

Patient satisfaction remained high, with scores of 98.09% in 2023 and 99.48% in 2024. These results are summarized in Figure 4, which provides a year-to-year comparison of satisfaction metrics, illustrating the consistency and potential improvement in perceived care quality.

### Fatigue assessment: IBD-F

**FIGURE 5:**
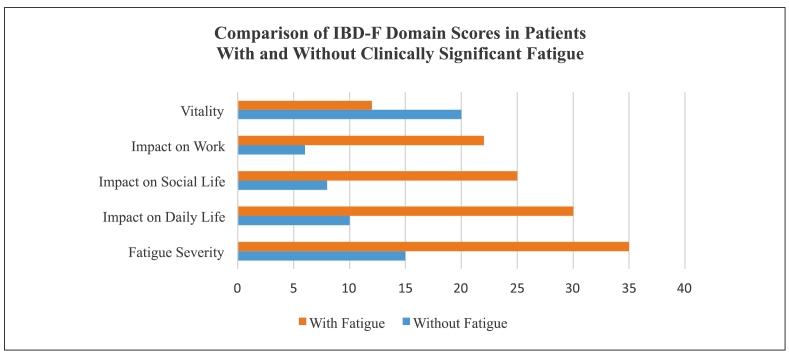
Comparison of IBD-F domain scores between patients with and without clinically significant fatigue. Source: authors.

The IBD-F was administered by the nurse to 32 patients in clinical remission. At the time of the survey, 53.1% reported no fatigue, while 59.5% had experienced moderate fatigue in the prior two weeks. Patients with fatigue showed markedly lower vitality and higher fatigue impact in all subdomains. Higher scores indicate greater fatigue severity (Figure 5). Fatigue impacted daily tasks in 37.4% and prompted naps in 46.8%; 37.5% reported QoL impairment.

### Mental health screening: HADS

**FIGURE 6:**
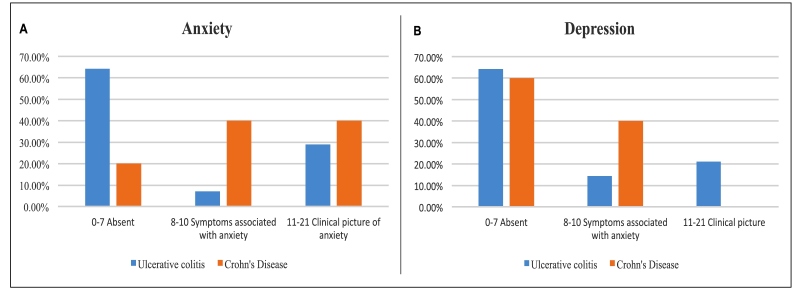
Evaluation of anxiety and depression A. Anxiety score. B. Depression score. Source: authors.

The HADS was self-administered by 90 patients under nurse supervision. Clinical anxiety was identified in 31.6% and clinical depression in 15.8%. Anxiety was more prevalent among CD patients (40%) (Figure 6A), while depression affected 21.4% of UC patients (Figure 6B). The nurse coordinated referrals to mental health services accordingly.

### Empowerment and research contribution

Empowerment was supported through structured education, interdisciplinary engagement, and research involvement. The nurse contributed to 41 research posters, including three award-winning presentations, and led patient education activities. Peer-led training and decision-making participation reinforced professional autonomy.

### Pediatric-to-adult transition

A structured internal tool was developed by the IBD nursing team to monitor adherence and psychosocial adaptation during the transition from pediatric to adult care. This tool consisted of six domains: treatment adherence, symptom awareness, emotional readiness, caregiver involvement, knowledge of disease and therapy, and patient autonomy. It was applied during pre- and post-transition visits. The complete instrument is available as SUPPLEMENTARY MATERIAL S2.

**Supplementary Material S2**

Pediatric-to-Adult Transition Monitoring Tool

This structured tool was developed by the IBD nurse team to evaluate patient readiness and follow-up during pediatric-to-adult transition. It includes 15 items grouped in six domains:

1. **Medication adherence** (e.g., “Do you take your medication as prescribed?”)

2. **Symptom knowledge and monitoring**

3. **Emotional readiness** (anxiety, fears, acceptance)

4. **Family/caregiver involvement**

5. **Knowledge of diagnosis and treatment**

6. **Autonomy and responsibility for self-care**

Each item is scored on a 3-point Likert scale (1 = never, 2 = sometimes, 3 = always). The tool was used to guide multidisciplinary discussions and tailor support during transition.

Ten pediatric patients transitioned to adult care under nurse coordination. The program included 3-5 multidisciplinary pre-transition visits and follow-up for 6-12 months. A structured internal tool supported adherence monitoring and psychosocial adaptation. Of these patients, 70% were female and 60% required biologics.

### Expanded nursing scope in IBD care

The nurse assumed responsibilities in:

- Fistulizing Crohn’s disease
- Ostomy and incontinence management
- Immunization and biologic therapy education
- Pregnancy, fertility, and lactation support
- Pain management and preventive care
- Nutritional counseling and sexual health guidance

These activities were documented via care logs and validated through clinical results, affirming the comprehensive and multidimensional role of nursing in IBD centers.

## DISCUSSION

This study highlights the integral and multifaceted role of nurses in the comprehensive management of patients with IBD at a Colombian Center of Excellence. Our findings emphasize how structured training, clinical coordination, education, research engagement, and telecommunication support collectively contribute to the operational and clinical success of IBD care models in Latin America.

The central role of the IBD nurse in care delivery-spanning phenotyping, symptom monitoring, transition care, education, and coordination-is consistent with international literature recognizing specialized IBD nursing as essential to improving healthcare quality^5^ ^-^ ^8^. Notably, our results illustrate that nurse-led interventions reached beyond conventional assistance, encompassing telemonitoring, organization of multidisciplinary meetings, and implementation of validated patient-reported outcome measures (PROMs), such as the IBDQ-32, IBD-F, and HADS scales. These tools allowed for the detection of quality-of-life impairments, fatigue, anxiety, and depression-critical aspects in IBD often underrecognized in routine consultations.

Although we did not measure clinical outcomes such as hospitalization or emergency visits, the implementation of PROMs, high patient satisfaction indicators, and structured follow-up processes suggest strong potential for improved patient-centered care. Accordingly, we adjusted the previously overstated claim about reduced hospitalizations to avoid unsupported inferences.

The nurse’s active participation in institutional quality improvement-through monitoring of adverse events, biologic therapy adherence, education coverage, and patient-reported indicators-demonstrates the capacity of nursing to contribute strategically to healthcare evaluation and planning. This aligns with prior studies that have shown how IBD nurses play a pivotal role in optimizing service delivery and supporting treatment continuity^6^ ^-^ ^8^.

Empowerment of nursing staff in this context is built upon a structured certification process, continuing education, peer-led teaching, and participation in research and patient education. While most initial training was physician-led, the integration of nurse-led modules and academic participation provided pathways toward professional autonomy, self-efficacy, and team leadership. This model may serve as a blueprint for IBD centers in Latin America where such roles remain underdeveloped.

Importantly, this study acknowledges the methodological challenge of potential bias in qualitative observations and interviews. To mitigate this, data collection followed standardized protocols and was triangulated through interviews, direct observation, and document review. Observers were trained to minimize influence on participants, and nurse involvement in data collection was separated from evaluation procedures to reduce expectation bias. These measures strengthen the validity of our findings while also reflecting the pragmatic challenges of observational research in real-world clinical environments.

Finally, this study reveals the underutilized potential of nurses to address critical areas in IBD care, including ostomy education, biologic therapies, pregnancy and fertility counseling, immunization, nutrition, and mental health. The structured pediatric-to-adult transition process led by the nurse-rarely reported in Latin American settings-offers a replicable model to ensure continuity and stability for young patients navigating between care systems.

This study has several limitations. First, it was conducted in a single IBD referral center, which may limit the generalizability of the findings to other institutions in Colombia or Latin America. Second, the analysis included only one certified IBD nurse, preventing comparisons across different professionals or care models. Third, the descriptive observational design does not allow causal inferences or the measurement of clinical outcomes such as hospitalization or relapse rates. Finally, although validated instruments (IBDQ-32, IBD-F, HADS) were used, patient-reported outcomes may be subject to recall or reporting bias. Despite these limitations, the study provides valuable exploratory insights into the essential role of nurses in IBD care.

## CONCLUSION

This research reinforces the essential contribution of specialized nurses to IBD multidisciplinary care. Through educational, clinical, emotional, and operational competencies, nurses enhance patient-centered care, contribute to institutional quality metrics, and bridge service gaps across complex care trajectories. Future multicenter and longitudinal studies should quantify the clinical impact of nurse-led interventions on outcomes such as disease control, healthcare use, and cost-effectiveness to further validate these roles in Latin America and beyond.

## Data Availability

Not applicable - The study did not use research data.
